# Whole-genome analysis of five *Escherichia coli* strains isolated from focal duodenal necrosis in laying hens reveals genetic similarities to the *E. coli* O25:H4 ST131 strain

**DOI:** 10.1128/spectrum.02110-24

**Published:** 2025-03-31

**Authors:** Yu-Yang Tsai, Julia Ienes Lima, Sonsiray Alvarez Narvaez, Catherine M. Logue

**Affiliations:** 1 Department of Population Health, College of Veterinary Medicine, University of Georgia 70734, Athens, Georgia, USA; 2 Department of Infectious Diseases, College of Veterinary Medicine, University of Georgia 70734, Athens, Georgia, USA; The Pennsylvania State University, University Park, Pennsylvania, USA

**Keywords:** *Escherichia coli*, avian, focal duodenal necrosis, disease, poultry

## Abstract

Focal duodenal necrosis (FDN) is an intestinal disease causing significant economic losses in the table-egg industry due to reduced egg production in laying hens. Its etiology and pathogenesis remain poorly understood. Between 2021 and 2023, 111 *Escherichia coli* isolates were collected from FDN lesions and screened for the presence of virulence genes using PCR panels. Five strains—FDN-4, FDN-9, FDN-11, FDN-24, and FDN-50—were selected for whole-genome sequencing due to their high virulence gene content. Core-genome analyses found that the five FDN *E. coli* belong to different phylogroups and strain types (ST), but they all share multiple complete operons involved in key pathogenic functions, including host cell adhesion and invasion, iron acquisition, motility, biofilm formation, and acid resistance. Comparative genomic analyses identified FDN-4 as the most genetically distinct strain, closely resembling EC958, an O25b:H4 ST131 uropathogenic *E. coli* (UPEC) commonly associated with extended-spectrum beta-lactamase production. FDN-4 and EC958 share unique chromosomal virulence genes absent in the other FDN strains, all located within genomic islands. This study provides the first complete genomic characterization of *E. coli* isolated from FDN lesions and highlights FDN-4 as a genetically distinct strain with similarities to O25b:H4 ST131 UPEC.

**IMPORTANCE:**

This study presents the first complete genomic characterization of *Escherichia coli* isolated from focal duodenal necrosis (FDN) lesions. Notably, FDN-4 is the first *E. coli* strain from a poultry disease (FDN) to show significant similarity to O25b:H4 ST131 strains, commonly classified as uropathogenic *E. coli* and often associated with extended-spectrum beta-lactamase production. However, caution is warranted when attributing direct transmission routes between poultry and humans.

## INTRODUCTION

*Escherichia coli* is one of the most extensively studied microorganisms in the world. Although *E. coli* is commonly found as part of the normal flora of the gastrointestinal tract in many animal species and humans (1), they are also recognized as one of the most significant pathogens causing a wide variety of diseases (2). *E. coli* is a species with diverse characteristics, and from a human health perspective, it can be classified into three main subsets: commensal strains, intestinal pathogenic *E. coli*, and extra-intestinal pathogenic *E. coli* (ExPEC) (3, 4). In avian species, avian pathogenic *E. coli* (APEC), as part of the ExPEC, has been reported to cause local and systemic infections in poultry, affecting a range of birds including chickens, turkeys, ducks, and others (1, 5).

Focal duodenal necrosis (FDN) is an intestinal disease that causes significant economic losses to the table-egg industry due to significant drops in egg production in laying hens (6). The etiology and pathogenesis of FDN are still poorly understood, but several research studies have indicated that *Clostridium perfringens* and *Clostridium colinum* may be associated with FDN even though a challenge model that fulfills Koch’s postulates has not been developed (6–8). Recently, our research group isolated *E. coli* from FDN lesions of laying hens (9). Through PCR analysis of virulence genes, we found some strains were APEC-like in nature but also carry several virulence genes present in *E. coli* strains involved with inflammatory bowel disease (IBD) in humans (9).

IBD has been associated with adherent invasive *E. coli* (AIEC) and includes the characterized strains LF82, HM605, NRG857c, and UM146 (10–14). Several studies indicate that there are genetic similarities between human *E. coli* (such as ExPEC) and APEC, particularly in the carriage of virulence genes (15, 16). Hence, it is possible that these avian *E. coli* isolates newly found causing FDN also share genetic similarities with other human-related strains. In this project, we used next-generation sequencing for the genomic characterization of five FDN-related *E. coli*. Our findings show that even when isolated from similar lesions, the tested *E. coli* isolates are genetically very different, belonging to different phylogenetic groups and harboring different plasmids.

## MATERIALS AND METHODS

### Sample collection and bacteria culture conditions

FDN lesions were placed in 200–300 µL of buffered peptone water (BD Difco, Franklin Lakes, NJ) and incubated at 37°C for 18–24 h both in aerobic and anaerobic conditions for bacterial enrichment. The FDN-lesion-containing broths were streaked onto tryptone soy agar (TSA) plates (BD Difco, Franklin Lakes, NJ) supplemented with 5% sheep blood (Remel, San Diego, CA) and MacConkey agar plates (BD Difco, Franklin Lakes, NJ). Plates were incubated at 37°C for 18–24 h both aerobically and anaerobically. Isolates suspected to be *E. coli* were inoculated into 1 mL of Luria-Bertani broth (BBL, Becton Dickinson, Franklin Lakes, NJ) and incubated at 37°C for another 18–24 h. Following incubation, bacteria were pelleted from the broth by centrifugation at 10,000 × *g* for 2 min in a 1.5 mL microtube centrifuge (Eppendorf, Germany). The pellet was resuspended in 200 µL of Ambion nuclease-free water (Life Technologies Corp., Austin, TX) and boiled at 100°C for 10 min in an Isotemp heating block (Fisher Scientific, Dubuque, IA). Once cooled at room temperature, the cell suspension was centrifuged at 10,000 × *g* for 2 min, and 180 µL of the supernatant containing bacterial DNA was transferred to a new tube and stored at –20°C until further use. Bacterial species determination was done with the 16S rRNA *E. coli* PCR as previously detailed (9). All *E. coli* strains were routinely maintained in brain heart infusion (BHI, BD Difco) broth with 20% glycerol and frozen at −80°C.

### Antimicrobial susceptibility testing

Antimicrobial susceptibility testing was performed using the National Antimicrobial Resistance Monitoring System (NARMS) panel (CMV4AGNF; Sensititre; ThermoFisher, Waltham, MA, USA) following the guidelines of the Clinical Laboratory Standards Institute (CLSI) (17). Isolates were initially cultured on TSA at 37°C for 24 h, after which colonies were transferred into 5 mL of sterile water and adjusted to a 0.5 McFarland standard. Then, 10 µL of the bacterial suspension was added to 11 mL of cation-adjusted Mueller Hinton broth with TES buffer, mixed thoroughly, and dispensed as 50 µL per well into a 96-well NARMS panel using the Sensititre AIM system (Thermo Fisher Scientific, Waltham, MA). The plates were sealed and incubated at 37°C for 18 h. MICs for each antimicrobial agent were determined using the Sensititre Optiread system (Thermo Fisher Scientific, Waltham, MA). MIC values and breakpoints were interpreted based on CLSI standards (M100) (17). Strains were categorized as resistant if their MIC was at or above the CLSI-recommended breakpoint for each antimicrobial. The breakpoints used in this study included ampicillin (≥32 µg/mL), amoxicillin-clavulanic acid (≥32/16 µg/mL), ceftriaxone (≥4 µg/mL), chloramphenicol (≥32 µg/mL), ciprofloxacin (≥1 µg/mL), trimethoprim-sulfamethoxazole (≥4/76 µg/mL), cefoxitin (≥32 µg/mL), gentamicin (≥8 µg/mL), nalidixic acid (≥32 µg/mL), sulfisoxazole (≥256 µg/mL), streptomycin (≥64 µg/mL), tetracycline (≥16 µg/mL), meropenem (≥4 µg/mL), and azithromycin (≥32 µg/mL). In cases where animal-specific breakpoints were unavailable, the human health breakpoints were applied.

### PCR panels for the detection of virulence genes

First, all *E. coli* isolates were run through the APEC pathotyping PCR panel, which covers the detection of APEC-related genes including *cvaC*, *iroN*, *ompT*, *hlyF*, *etsB*, *iss*, *aerJ*, *ireA*, and *papC* (9, 18). Then, the presence of IBD-related genes in the isolates was determined using an IBD PCR panel that included the genes *fimH*, *dsbA*, *eaeH*, *fepC*, *ratA*, *colV*, *usp*, and *irp2* (9, 19, 20). The PCR primers and protocol for colicin (E1, B, Ia, and M) detection and microcin (mV, mH47, and mM) were described by Gordon et al. (21). IBD virulence determinants *ial*, *aer*, *pap*, and *cnf1* were PCR tested using primers and conditions described by Birosova et al. and López-Saucedo et al. (*22*). All amplification products were analyzed by gel electrophoresis in 2% SeaKem LE Agarose (Lonza, Rockland, ME) followed by staining in 0.25% ethidium bromide (Thermo Scientific, Rockford, IL) solution for 20 min and viewed under UV light using UVP BioDoc-It 2 Imager (analytikjena, Upland, CA).

### Genomic library preparation and raw read processing

Five *E. coli* strains were selected and grown in BHI at 37°C for 24 h. The cell suspension was centrifuged at 10,000 × *g*, and the cell pellet was collected once the supernatant was removed. The cell pellet was shipped to Mr. DNA (www.mrdnalab.com, Shallowater, TX, USA) for whole-genome sequencing (WGS) and raw read processing. On arrival, the bacterial pellet of each strain was re-suspended in 180 µL of ATL buffer (Qiagen, Germantown, MD) and was used to extract the high molecular weight DNA using the MagAttract HMW DNA Kit (Qiagen, Germantown, MD). The DNA concentration was evaluated using the Qubit dsDNA HS Assay Kit (Life Technologies, Carlsbad, CA), and sample quality was determined using a NANODROP 2000 (ThermoFisher Scientific, Waltham, MA). The size of each gDNA sample was determined using electrophoresis (E-Gel SizeSelect 2% Agarose Gel; Invitrogen), which confirmed a fluorescent band >15 Kb, respectively, for each sample. The samples were then sheared using the Covaris G-tube (Covaris Inc., Woburn MA), and the average size of each sample was verified using the Agilent 2100 Bioanalyzer (Agilent Technologies, Santa Clara, CA). Approximately 1–1.5 µg of gDNA was used as input for library preparation using SMRTbell Express Template Prep Kit 2.0 (Pacific Biosciences, San Diego, CA). Briefly, the samples underwent DNA damage repair, end repair, and barcode adapter ligation. After ligation, libraries were pooled in equimolar concentrations with 5–6 libraries per pool. Unligated DNA fragments were removed using a nuclease treatment, and following library preparation, DNA fragments >7 Kb were selected for sequencing using the BluePippin automated size-selection instrument (Sage Science Inc., Beverly, MA). The final library concentration was measured using the Qubit dsDNA HS Assay Kit (ThermoFisher Scientific Waltham, MA), and the average library size was determined using the Agilent 2100 Bioanalyzer (Agilent Technologies, Santa Clara, CA). The final library was then sequenced using the 30 h movie time on the PacBio Sequel IIe (Pacific Biosciences, Menlo Park, CA) sequencer.

HiFi genome assembly was accomplished using the Improved Phase Assembler (IPA) via SMRT Link 10.1.0. The IPA consists of several key processes including overlapping (pancake), phasing (nighthawk), filtering overlaps (falcon), contig construction (falcon), and polishing (racon). All assembly tools were obtained from PacBio (www.pacb.com) and used at the default setting unless otherwise mentioned. The genomes of the five sequenced *E. coli* were deposited in the National Center for Biotechnology Information (NCBI) database under accession numbers: CP158026.1 (*E. coli* FDN-4), CP158140.1 (*E. coli* FDN-9), CP158143.1 (*E. coli* FDN-11), CP158147.1 (*E. coli* FDN-24), and CP158150.1 (FDN-50; Table S1).

### Core genome analysis

Basic genomic information, such as chromosome size, coding DNA sequences (CDS), GC content, and tRNA, was obtained using Geneious Prime 2024.0.3 (https://www.geneious.com) and the Bacterial and Viral Bioinformatics Resource Center web resources (https://www.bv-brc.org/) (23). The virulence genes were identified through ABRicate v1.0.1 (https://github.com/tseemann/abricate) using the vfdb (http://www.mgc.ac.cn/VFs/) and ecolivf databases. The gene presence was considered only with a coverage/identity greater than 90%/80%. Antimicrobial resistance (AMR) genes were identified using AMRFinder Plus v3.9.8 (24) and ResFinder database from ABRicate (accessed on 5 July 2023). All isolates were *in silico* typed with regard to *E. coli* O:H serotypes and sequence type (ST) using the programs and the database of the Center for Genomic Epidemiology (http://www.genomicepidemiology.org/services/), specifically SerotypeFinder with 85% threshold for minimum identity and 60% minimum length (25) and MLST 2.0 (26–32). *E. coli* phylogenetic type was determined using the EzClermont program, available as a web app at www.ezclermont.org (33).

In addition to five FDN strains, the O25b:H4 ST131 representative strain EC958 (#NZ_HG941718.1) was included in the following analysis. Genome annotation was conducted using Prokka v.1.14.6 with a similarity *e*-value cut-off of 1e-6 (34), and the output files were submitted to Roary v.3.13.0 (35) using default parameters. The gene presence/absence table generated by Roary was used as the input file in Scoary v.1.6.16 (36) with a *P*-value cut-off of 0.05, adjusted via the Bonferroni correction, to establish gene clusters associated with each trait. The chromosomes of the five FDN strains were aligned to IBD-related strains HM605, LF82, and UM146 using the nucleotide Basic Local Alignment Search Tool (BLASTn) from the NCBI (https://blast.ncbi.nlm.nih.gov/Blast.cgi). A whole-genome alignment circular plot image was generated with GView (37) using the default settings of the software. When generating the plot, FDN-4 was placed in the center of the rings as the reference strain and strain EC958 as the outermost ring (38). Additionally, core genomes were used to construct a maximum likelihood phylogenetic tree using CSI Phylogeny 1.4 (https://cge.food.dtu.dk/services/CSIPhylogeny/) (39), with the core genomes included in this analysis as shown in Table S1. The resulting tree was visualized using the online Interactive Tree of Life platform (https://itol.embl.de).

### Accessory genome analysis

Plasmids were identified using PlasmidFinder 2.1 and the database of the Center for Genomic Epidemiology with a 95% threshold for minimum identity and 60% minimum coverage (32, 40). The virulence genes carried by plasmids were also identified through ABRicate v1.0.1 (https://github.com/tseemann/abricate) using the vfdb (http://www.mgc.ac.cn/VFs/) and ecolivf databases. A gene was considered present only with a coverage/identity greater than 90%/80%.

Genomic island (GI) identification was conducted using IslandViewer4 (https://www.pathogenomics.sfu.ca/islandviewer/), which integrates IslandPath-DIMOB, SIGI-HMM, and IslandPick to provide the most comprehensive prediction of genomic islands (41). Since multiple methods are integrated by IslandViewer4, when determining the start and end positions of GIs, sometimes these GI predictions overlap. When this occurred, the overlapping GIs were combined. The circular plot showing genomic islands was downloaded from the tool for further analysis. BLASTn was used to identify GIs and determine if they were present in other *E. coli* strains (Table S2).

## RESULTS

### IBD-related genes are routinely found in *E. coli* isolated from FDN lesions in laying hens

A total of 72 *E. coli* isolates were collected from FDN lesions in laying hens between 2021 and 2023. Of these, 24 (33.3%) possessed at least four APEC-related virulence genes (including *ompT* and *hlyF* genes) and were considered APEC-like strains. Additionally, 19 different IBD-related genes were detected among all tested *E. coli* isolates, with the most frequently detected genes being *dsbA*, *fimH*, *eaeH*, and *fepC* (Table S3). When incorporating the FDN-related *E. coli* isolated in our previous study (*n* = 39), 88 out of the 111 tested strains (79.3%) carried these four genes, and at least four of the 19 IBD-related genes were found in 93 isolates (83.8%), with 20 strains (18%) harboring nine or more (Table S3).

Four strains—FDN-9, FDN-11, FDN-24, and FDN-50—in which more than 17 genes (IBD and Patho) were detected from a previous study were subjected to WGS, together with *E. coli* FDN-4 that carried 14 IBD-related genes in this study (9) (Table 1). Antimicrobial susceptibility testing was performed on the five FDN *E. coli* and determined that they were susceptible to all antimicrobials tested (Table S4).

**TABLE 1:** Result of IBD and Patho PCR panels in five FDN strains examined^*a*^

	Gene strain	FDN-4	FDN-9	FDN-11	FDN-24	FDN-50
IBD-related genes	*eaeH*	+	+	+	+	+
	*irp2*	−	−	−	−	−
	*fimH*	+	+	+	+	+
	*ratA*	−	−	−	−	−
	*fepC*	+	+	+	+	+
	*usp*	+	−	−	−	−
	*colV*	−	−	−	−	−
	*dsbA*	+	+	+	+	+
	*ial*	+	+	+	+	+
	*aer*	+	−	−	−	−
	*cnf1*	−	−	−	−	−
	*pap*	−	−	−	−	−
	Colicin E1	+	+	+	+	−
	Colicin B	+	+	+	+	+
	Colicin Ia	−	+	−	+	−
	Colicin M	−	−	+	−	+
	Microcin mV	−	+	+	+	+
	Microcin mH47	−	+	+	+	+
	Microcin mM	−	+	−	+	+
Subtotal		9	11	10	11	10
Patho genes	*cvaC*	−	+	+	+	+
	*iroN*	+	+	+	+	+
	*ompT*	+	+	+	+	+
	*hlyF*	+	+	+	+	+
	*etaB*	+	−	−	−	−
	*iss*	+	+	+	+	+
	*aerJ*	−	+	+	+	+
	*ireA*	−	−	+	−	+
	*papC*	−	−	−	−	−
Subtotal		5	6	7	6	7
Total (IBD + Patho)		14	17	17	17	17

^*a*^ “+” indicates plasmid positive for the targeted gene; “-” indicates plasmid negative for the targeted gene.

### Characterizing the core genome of IBD-related FDN *E. coli*

**Fig 1:**
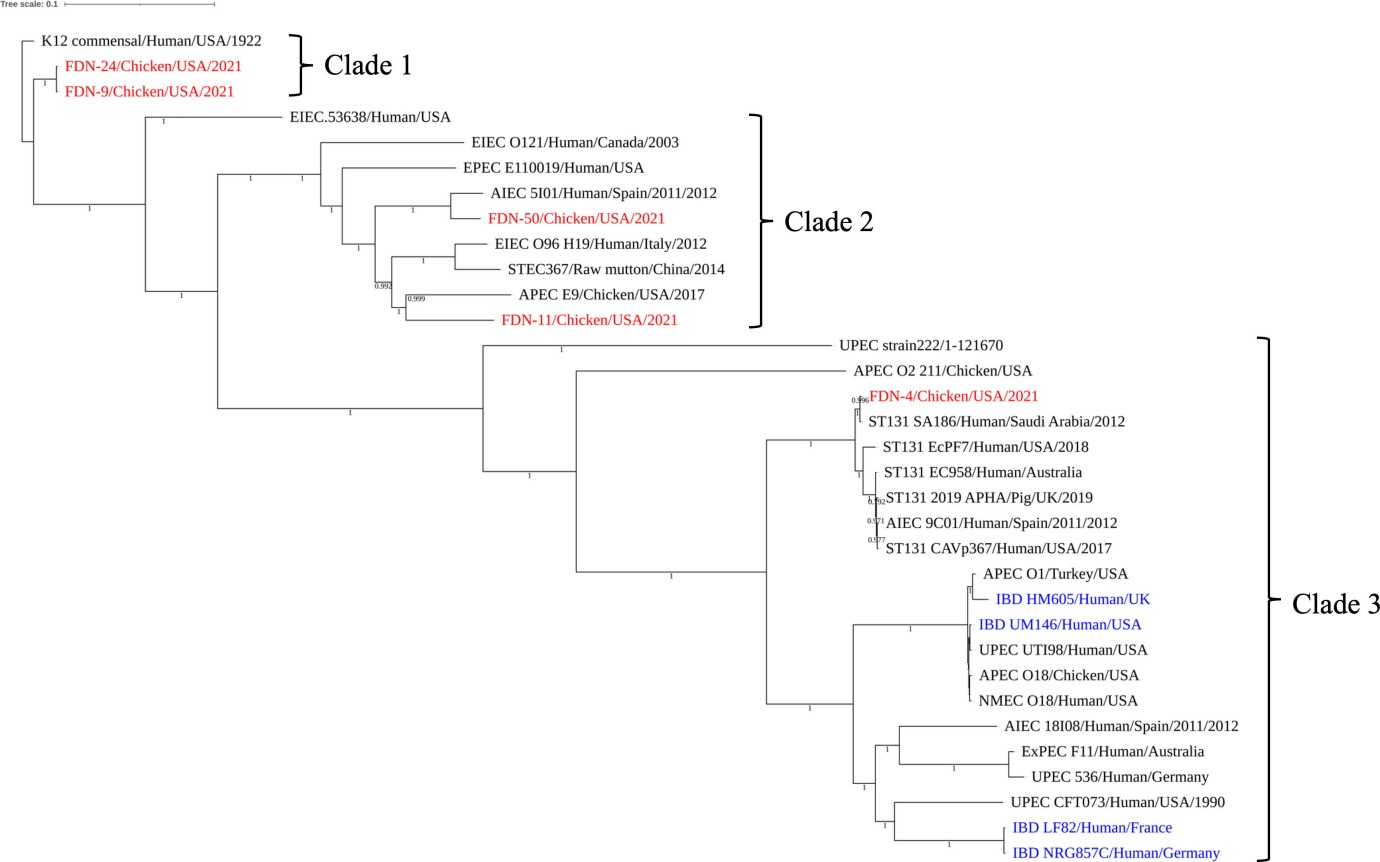
Phylogenetic tree built with *E. coli* sequences shows the host, country, and year of isolation.

The chromosome size of the five sequenced *E. coli* genomes ranged from 4,797,640 bp (*E. coli* FDN-24) to 5,149.337 bp (*E. coli* FDN-4), and they all shared a similar GC content (50%; Table 2). When their chromosomes were compared, FDN-4 was found to be the most distant from the other four FDN isolates with the lowest percent query cover and percent identity (Table S5). On the contrary, FDN-9 and FDN-24 have an almost identical genome (sharing 99% query cover with 100% identity) and were classified in the same serotype (H32 and O26), strain type (ST10), and *E. coli* phylotype (type A). All the other isolates were classified as different serotypes and strain types, although FDN-11 and FDN-50 shared the same phylogenetic type (type B1; Table 2). FND-4 was found genomically very similar to IBD-related *E. coli* HM605, LF82, NRG857c, and UM146 (query cover <83% with a percent identity <98.91%) (Table S5). Notably, a core genome SNP-based phylogenetic analysis placed FDN-4 among *E. coli* IBD strains, ST131 uropathogenic *E. coli* (UPEC) strains, and other pathogenic representatives from UPEC, AIEC, APEC, and neonatal meningitis-causing *E. coli* (NMEC; Fig. 1). The same analysis placed FDN-9 and FDN-24 together in a clade that contained *E. coli* K-12 commensal strain, while FDN-11 and FDN-50 grouped with APEC, AIEC, enteropathogenic *E. coli* (EPEC), enteroinvasive *E. coli*, and enterohemorrhagic *E. coli* (Shiga toxin-producing *E. coli* [STEC]) strains. These results provide evidence that FDN-related *E. coli* is genetically very diverse even when recovered from similar lesions.

**TABLE 2:** Genome, plasmid information, phylogenetic type, H type, O type, and ST of the five FDN strains sequenced

	Chromosome size (bp)	CDS	GC content (%)	tRNA	# Plasmids	Phylogenetic type	H type	O type	ST	Plasmid name	Plasmid size (bp)	Plasmid type
FDN-4	4,966,509	4,972	50.69	87	3	B2	H4	O25	ST131	pFDN4-1	182,828	IncFIB/IncFIC
										pFDN4-2	35,593	IncX1
										pFDN4-3	7,419	Not identified
FDN-9	4,798,160	4,872	50.5	86	2	A	H32	O26	ST10	pFDN9-1	148,663	IncFIB/IncFII
										pFDN9-2	88,248	IncI1-I
FDN-11	4,845,341	4,845	50.61	88	3	B1	H8	O88	ST101	pFDN11-1	135,559	IncFIB/IncFIC
										pFDN11-2	48,395	Not identified
										pFDN11-3	32,596	IncX1
FDN-24	4,797,640	4,873	50.5	86	2	A	H32	O26	ST10	pFDN24-1	148,663	IncFIB/IncFII
										pFDN24-2	88,172	IncI1-I
FDN-50	4,921,440	5,067	50.8	87	3	B1	H51	O86	ST155	pFDN50-1	158.974	IncFIB/IncFII
										pFDN50-2	89,593	IncI1-I
										pFDN50-3	36,542	IncX4

Our *in silico* virulence factor identification determined that *E. coli* FDN-4 carried the most virulence genes in its chromosome (*n* = 191), followed by FDN-50 (*n* = 187), FDN-11 (*n* = 165), and FDN-9/FDN-24 (both with *n* = 156 genes; Table S6.1). A total of 103 virulence factors were shared by all five FDN *E. coli*, including genes and operons responsible for host-cell adhesion (*csgABCDEFG*, *fimABDFGHI*, *ecpABCDER*, *eaeH*, *fdeC*, and *ompA*), iron acquisition (*entABCDEFS*, *fepABCDG*, *fes*, and *flk*), flagella/motility and biofilm formation (*flgABCDEFGHIJKLN*, *flhABCDE*, and *fliAEFGHIJKLMNOPQRSTYZ*), acid resistance (*gadX*), and host-cell invasion (*ibeB*; Table S6.2). Meanwhile, there were 50 genes present in FDN-4 and EC958 but not in other FDN strains that include 16 genes and operons associated with iron acquisition (*chuASTUVWXY* and *ybtAEPQSTUX*), capsule formation (*kpsDMT* and *neuAC*), host-cell adhesion (*pixABCDFGHJ* and *matAC*), invasion (*tia*), and genotoxin production (*usp*; Table S6.3).

In addition to virulence factors, the multidrug resistance (MDR) gene *mdf(A)_1* was found in all the chromosomes of the FDN *E. coli*, and strain FDN-4 also presented chromosomal mutations *ptsI_*V25I and *uhpT_*E350Q that confer fosfomycin resistance.

### Characterizing the accessory genome of IBD-related FDN *E. coli*

All FDN *E. coli* isolates carried plasmids. FDN-4 and FDN-50 harbored three plasmids, while isolates FDN-9, FDN-11, and FDN-24 carry two plasmids each (Table 2). The plasmids identified belong to incompatibility groups *IncF*, *IncI*, and *IncX* with sizes ranging from 7,419 to 158,974 bp. All the plasmids have been previously identified in other bacteria, primarily in *E. coli* but also in *Klebsiella pneumoniae* and *Salmonella enterica*. The range of host species in which these plasmids were found is very broad and includes humans, other mammals (sheep, giant panda, and bovine), and avian species (gull and chicken) worldwide (Table S7). Notably, the plasmid profiles of the FDN *E. coli* are all different except for strains FDN-9 and FDN-24 which share identical plasmids.

Several virulence factors carried by plasmids appeared consistently in FDN-4, FDN-9, FDN-11, and FDN-24. Among them, the siderophore-related *iroBCDEN*, *iucABCD*, and *sitABCD* gene clusters are functionally related in providing a comprehensive system for siderophore-mediated iron uptake (Table 3). The plasmids of *E. coli* FDN-4 also harbor extra virulence genes *aatA* (amino acid transport and metabolism) and *tsh* (temperature-sensitive hemagglutinin) that play roles in the adhesion or secretion of virulence factors. Notably, *tsh* is present in the GI (GI39 and GI40). The plasmids of *E. coli* FDN-9 and FDN-24 also possess genes involved in plasmid maintenance and stability (*ccdB* and *traJ*) and pathogenicity (*astA* and *cvac*). Only two additional virulence genes regarding bacterial invasion (*ibeCI*) and conjugation control (*trat*) were observed in the plasmids of FDN-50 (Table 3). No AMR genes were identified in any of the FDN plasmids, corroborating the susceptible phenotype of the strains as found by the NARMS analysis.

**TABLE 3:** Virulence genes detected in plasmids in each FDN strain^*a*^

	pFDN4-1	pFDN4-3	pFDN9-1	pFDN9-2	pFDN11-2	pFDN24-1	pFDN24-2	pFDN50-1
*aatA*	+	-	-	-	-	-	-	-
*astA*	-	-	-	+	-	+	-	-
*cba*	-	-	-	+	+	+	-	-
*ccdb*	-	-	-	+	+	+	-	-
*ce1a*	-	+	-	-	-	-	-	-
*cia*	-	-	+	-	-	-	+	-
*cma*	+	-	-	-	+	-	-	-
*cvac*	-	-	-	+	+	+	-	-
*hbp*	-	-	-	-	+	-	-	-
*ibeC*	-	-	-	-	-	-	-	+
*iroB*	+	-	-	+	+	+	-	-
*iroC*	+	-	-	+	+	+	-	-
*iroD*	+	-	-	+	+	+	-	-
*iroE*	+	-	-	+	+	+	-	-
*iroN*	+	-	-	+	+	+	-	-
*iss2*	+	-	-	+	+	+	-	-
*iucA*	+	-	-	+	+	+	-	-
*iucB*	+	-	-	+	+	+	-	-
*iucC*	+	-	-	+	+	+	-	-
*iucD*	+	-	-	+	+	+	-	-
*iutA*	+	-	-	+	+	+	-	-
*mchf*	-	-	-	+	+	+	-	-
*sitA*	+	-	-	+	+	+	-	-
*sitB*	+	-	-	+	+	+	-	-
*sitC*	+	-	-	+	+	+	-	-
*sitD*	+	-	-	+	+	+	-	-
*traJ*	-	-	-	+	-	+	-	-
*trat*	+	-	-	+	+	+	-	+
*tsh*	+	-	-	-	-	-	-	-
Total	19	1	1	22	22	22	1	2

^*a*^ “+” indicates plasmid positive for the targeted gene; “-” indicates plasmid negative for the targeted gene.

### GIs, key components of the genomic diversity of *E. coli* FDN-4

**Fig 2:**
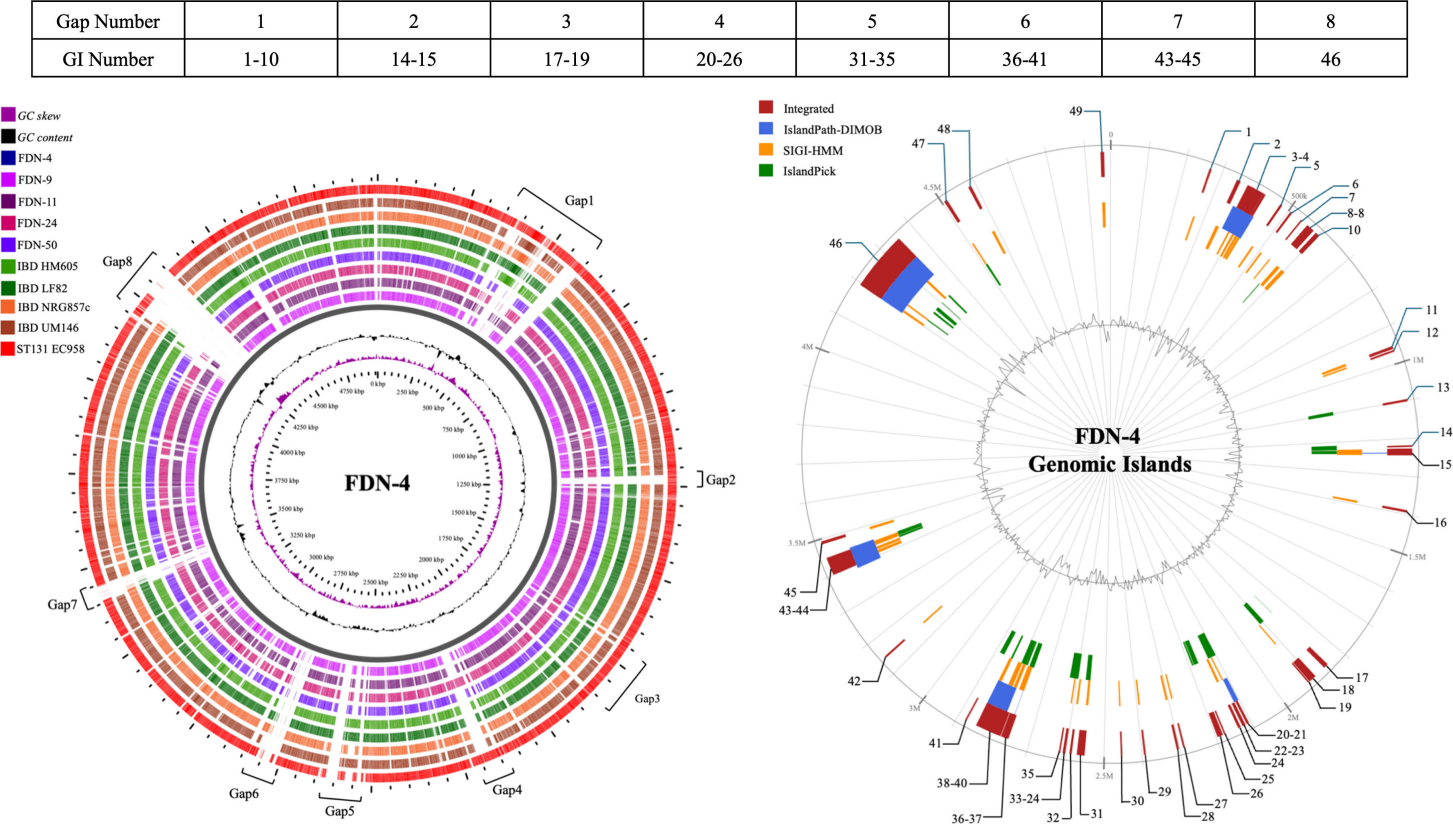
Two circular plots comparison analysis of whole-genome alignment (left) and GI (right). The table shows the corresponding gap to the location of GIs.

Figure 2 illustrates whole-genome alignment across different *E. coli* strains with FDN-4 as a reference. Significant gaps in the alignment indicate FDN-4 genome segments that consistently differ from other strains. Notably, the outermost red ring (EC958) appears the most complete, suggesting a higher degree of similarity with FDN-4 compared to other strains. The circular plot on the right maps the distribution of genomic islands within the FDN-4 chromosome. By comparing the two plots, it is evident that several alignment gaps correspond directly to the locations of genomic islands. For instance, gap 1 aligns with GIs 1–10, gap 2 with GIs 14–15, and gap 3 with GIs 17–19. Similarly, other gaps match with specific GI clusters as shown in the accompanying table (Fig. 2). A total of 49 GIs were identified spread across the chromosome of *E. coli* FDN-4, with sizes ranging from 4,001 bp to 157,890 bp (Fig. 2; Table S2). Among these GIs, 40 (81.6%) are also present in ExPEC strain ST131 O25:H4, while the rest (*n* = 4, 18.4%) have been identified in UPEC, extended-spectrum beta-lactamases (ESBL)-producing *E. coli*, APEC, STEC, and colistin-resistant *E. coli*. Furthermore, the individual genes and operons shared by FDN-4 and EC958 were found to be part of GIs (Table 4; Table S8). The GIs that contain most of these genes are GI 46 (*n* = 14) and GI 39 (*n* = 10). Some of the genes included in GI 46 are *bfpB* (pili biogenesis), *cirA_5* (iron uptake), *dnaB* (DNA helicase), *dpnM* (DNA methylase), *hin* (DNA recombinase), *klcA_1* (anti-restriction), *rcbA* (genome stability), *rep_2* (DNA replication), *ssb* (ssDNA binding), *tcpE* (pilus-related), *tfaE* (tail fiber assembly), *topB_2* (topoisomerase), *xerC* (DNA recombinase), and *ykgM_2* (ribosomal protein). Some of the genes present in GI 39 include *der* (ribosome biogenesis), *espC* (autotransporter protease), *fimACD* (fimbriae assembly), *klcA_1* (anti-restriction), *papACDFH* (*P* fimbriae), *pdeIL* (phosphodiesterase), *tsh* (hemagglutinin autotransporter), *virF* (virulence regulator), *yjdJ_2* (stress response), and *yraIJ* (virulence-associated).

**TABLE 4:** The genes/gene clusters present only in FDN-4 and EC958 and their location in the GI and the corresponding gap number when comparing with whole-genome alignment circular plot

Gap number	GI number	Genes
1	2	*dapAH*, *mshA_2*, *rfbC*, and *wbbL*
	3	*cirA_5*, *klcA_1*, *RBKS_2*, and *xerC*
	8	*dnaB* and *xerC*
	9	*ybcO*
	11	*fimACD*
2	14	*intAS*
	15	*xerC*
3	17	*aplK*, *aptAB*, *fabG_2*, *gdh*, *rbsABC*, and *sorC_1*
4	20	*xerC*
	21	*xerC*
	24	*ykgM_2*
	25	*fliD_1*
	26	*fliD_1* and *tagD_2*
	27	*fimACD* and *yraIJ*
	28	*yadCNV*
	29	*dapAH* and *RBKS_2*
5	32	*xerC*
	33	*ykfI*
	34	*der*
6	36	*intAS*, *pagN*, and *yhbX*
	37	*cra*, *hns_2*, *intAS*, *pagN*, *sacX*, *scrBKY*, and *yhbX*
	39	*der*, *espC*, *fimACD*, *klcA_1*, *papACDFH*, *pdeIL*, *tsh*, *virF*, *yjdJ_2*, and *yraIJ*
	40	*fimACD*, *tsh*, *virF*, and *yraIJ*
7	43	*der*, *hsdS*, *klcA_1*, *pagN*, *proQ_2*, and *thyA_1*
	44	*hsdS*, *intAS*, *pagN*, and *yhbX*
	45	*rfaBP*
8	46	*bfpB*, *cirA_5*, *dnaB*, *dpnM*, *hin*, *klcA_1*, *rcbA*, *rep_2*, *ssb*, *tcpE*, *tfaE*, *topB_2*, *xerC*, and *ykgM_2*
	48	*cra*, *sacX*, *scrBKY*, and *yqcG*
	49	*yfdC*

## DISCUSSION

From 2021 to 2023, our studies consistently identified several IBD-related genes in *E. coli* isolates from FDN lesions. The most frequently detected genes were *dsbA*, *eaeH*, *fimH*, and *fepC*. This finding aligns with our previous study, which also identified these four genes as the most prevalent in strains from FDN samples (9). Their functions may partially explain the potential role of these genes in causing FDN lesions in the chicken duodenum. For example, the *eaeH* gene is associated with adhesion and colonization of the small intestine in humans, while *fimH* plays a key role in bacterial adhesion to host cells, particularly in urinary tract infections (UTIs) and gut colonization (42, 43). Gene *fepC* is essential for *E. coli* to acquire iron and *dsbA* catalyzes the formation of disulfide bonds in proteins such as adhesins, toxins, and other virulence-associated factors, making it essential for bacterial pathogenicity (44, 45).

The consistent detection of IBD-related genes in FDN *E. coli* strains through the years encouraged us to pursue a deeper genomic characterization of these strains. We selected five representative isolates that had the highest number of virulence genes detected via PCR for further study: FDN-4, FDN-9, FDN-11, FDN-24, and FDN-50. FDN-4 was identified as a representative of phylogenetic group B2, which is the phylogenetic group associated with extraintestinal disease-causing strains (46), and it was classified as serotype O25:H4 ST131, a lineage linked to human UTIs (47). In the produced phylogeny, FDN-4 grouped with human IBD strains and ST131 *E. coli*, including AIEC, UPEC, and APEC strains. When we group FDN-4 with other ST131 strains, we found it is most closely related to strain SA186 from humans in Saudi Arabia (Fig. S1). FDN-9 and FDN-24 were identified as belonging to phylogenetic group A, which is generally considered less pathogenic than strains from phylogenetic groups B2 or D but can still cause disease under certain circumstances (48). These two FDN isolates share identical core genomes and are closely related to the *E. coli* K-12 strain. FDN-11 and FDN-50 were classified as phylogenetic group B1, which primarily includes commensal strains(49), but also contains the AIEC 5I01 strain isolated from a patient with Crohn’s disease (50) and the recently reported MDR APEC strain E9 (51). In fact, our phylogenetic analysis clusters FDN-11 and FDN-50 with AIEC strain 5I01 and APEC strain E9, indicating potential pathogenic overlap (50, 51). Overall, these results provide evidence that FDN-isolated *E. coli* exhibit significant genetic diversity, even when recovered from the same lesions.

All five FDN *E. coli* shared numerous complete operons in their chromosomes involved in key pathogenic functions, including host-cell adhesion and invasion, iron acquisition, motility, biofilm formation, and acid resistance. Among the adhesion-related genes, the *csg* and the *fim* operons and *ompA* gene have been found in IBD-associated *E. coli* (52–54). The *csg* and *fim* operons encode curli and type 1 fimbriae, respectively, facilitating bacterial adhesion and biofilm formation (55, 56). OmpA, also found in other enteropathogenic bacteria like *Enterobacter sakazakii*, helps in epithelial invasion and has been implicated in intestinal infections (57). The *eaeH* gene encodes a variant of the intimin protein, which may contribute to epithelial attachment and effacement lesions, similar to EPEC (43). Additionally, the *fdeC* gene, an autotransporter adhesin, supports *E. coli* colonization in both avian and mammalian hosts (58, 59). Iron acquisition is essential for bacterial survival in iron-limited environments. The *ent*, *fep*, *fes*, and *flk* operons enable iron uptake, with *fep* specifically associated with intestinal pathogenicity (60). Given the importance of iron acquisition in APEC, these operons likely enhance the survival and virulence of FDN *E. coli* (61, 62). Motility and initial surface attachment are mediated by the *flg*, *flh*, and *fli* operons, which encode flagellar components essential for movement and colonization and contribute to biofilm formation (63–65). Finally, *ibeB*, a gene linked to bacterial invasion, is known to enhance the colonization of multiple organs, including the brain and lungs, in APEC strains (66). Overall, the mentioned genes and operons, present in all FDN *E. coli* strains, likely drive the pathogenicity of FDN *E. coli* and contribute to the intestinal lesions characteristic of the disease.

FDN-4 was found to be the most unique among the FDN strains. This difference may be due to virulence genes within GIs that are also present in human isolate EC958. This suggests that FDN-4 may have acquired them through horizontal gene transfer (HGT) from the ST131 strain. GI 46 and GI 39 harbor key virulence-associated genes that enhance FDN-4’s pathogenic potential. GI 46, which contains the highest number of virulence factors, includes housekeeping genes (*dnaB*, *rep_2*, *ssb*, *topB_2*, and *xerC*) that maintain the GI during HGT and drive genetic diversity (67–70). It also encodes *bfpB*, essential for pilus-mediated adherence, and *cirA_5*, which supports bacterial growth by facilitating iron acquisition in iron-limited environments (71, 72). Additionally, genes like *hin*, *tfaE*, and *tcpE* enhance adaptability by modulating antigen expression, mobilizing phage-related elements, and facilitating gene transfer (73–75). GI 39 contains 10 virulence-related genes that contribute to host colonization and immune evasion. Adhesion factors like *fimACD* and *papACDFH* operons promote attachment to urinary and intestinal tissues, while *espC* and *tsh* induce cytotoxic and proteolytic damage (76–79). VirF regulates virulence gene expression in response to host cues, optimizing resource use and avoiding host immune detection/response (80, 81). Metabolic genes (*der* and *yjdJ_2*) support bacterial growth, protein synthesis, and stress resistance, enabling FDN-4 to persist and outcompete native microbiota (82, 83). Together, these traits suggest that GIs enhance FDN-4’s ability to establish infection, evade host immune defenses, and adapt to diverse host environments.

Many of the virulence factors unique to the FDN-4 genome, absent in other FDN-related *E. coli*, are also found in EC958, the representative strain of the highly pathogenic O25:H4-ST131 lineage, potentially enhancing FDN-4’s pathogenicity (47). Notably, FDN-4 harbors the *chu* and *ybt* iron acquisition systems, distinguishing it from other FDN strains. While *chu* is crucial for UPEC virulence, *ybt* in AIEC is linked to intestinal fibrosis in IBD models (84–86). Capsule biosynthesis genes *kps* and *neuAC*, exclusive to FDN-4, facilitate immune evasion by inhibiting phagocytosis and complement-mediated killing (87–90). These genes also enhance APEC colonization in the chicken intestine, potentially contributing to intestinal lesions in FDN-affected duodenums (91). Other independently functional genes include *tia*, which promotes epithelial invasion in STEC and APEC, and *usp*, a genotoxin linked to UPEC and detected in *E. coli* from chicken meat (92–95). The *pix* fimbrial gene supports UPEC adhesion, aiding in urinary tract infections (96, 97). Overall, FDN-4 exhibits greater similarity to ExPEC strains like UPEC and NMEC, with genes that enhance immune evasion, persistence, and host colonization.

*E. coli* O25:H4 ST131 is commonly found carrying fluoroquinolone-resistance and ESBL resistance traits (98, 99). These strains contribute significantly to the global rise of *E. coli* uropathogens producing CTX-M ESBLs and were pivotal in the 2016 “CTX-M pandemic” (100, 101). Most of the AMR genes in *E. coli* O25:H4 ST131 are carried by MDR plasmids, such as pEK499, pEK516, and pEK204 (102). FDN-4 does not possess such plasmids, making it susceptible to all the antibiotics included in the NARMS panel. The presence of two point mutations associated with fosfomycin resistance, *ptsI_V25I* and *uhp_E350Q*, in the chromosome of FDN-4, but absent in other FDN or IBD strains, is noteworthy. Fosfomycin is commonly employed in treating UTIs in humans (103). EC958 is a common UTI pathogen; hence, it is not surprising to find that this MDR strain develops resistance to fosfomycin (38). The fact that FDN-4 shows this resistance genotype is intriguing and further supports the similarity between FDN-4 and the UPEC ST131 strains (104).

### Conclusions

This study provides the first complete genomic characterization of APEC isolated from FDN lesions. Through virulence gene profiling, phylogenetic analysis, and genomic island comparisons, we demonstrated that one of the recovered APEC strains, FDN-4, shares significant genetic features with EC958, a representative ST131 strain. The detection of ST131-like strains in poultry suggests a potential role for birds as reservoirs of these bacteria (105, 106). However, caution is needed when attributing direct transmission routes between poultry and humans. Unlike typical ST131 strains, FDN-4 lacks MDR plasmids and is susceptible to all major antimicrobials tested. It is important to note that the presence of FDN-4 in the intestinal lesions does not establish its role in the etiology of the disease since *E. coli* belonging to other phylogroups and STs has been equally isolated from the lesions, and the pathogenesis of FDN is still under investigation. To gain a clearer understanding, it is crucial to gather and analyze additional *E. coli* from FDN samples and elucidate the connection between this ST131 strain and the disease, as well as any potential implications for public health.

## SUPPLEMENTAL MATERIAL

Figure S1 spectrum.02110-24-s0001.docx Phylogenetic tree with FDN-4 and O25:H4 ST131 strains from the Enterobase database.

Table S1 spectrum.02110-24-s0002.docx *E. coli* strains included in the phylogenetic tree.

Table S2 spectrum.02110-24-s0003.xlsx Genomic island information and the genes present with their products in FDN-4.

Table S3 spectrum.02110-24-s0004.xlsx Heatmap of PCR results from the IBD and Patho panels in *E. coli* samples isolated from FDN lesions between 2021 and 2023.

Table S4 spectrum.02110-24-s0005.xlsx Results of antimicrobial susceptibility testing by NARMS panel in FDN strains.

Table S5 spectrum.02110-24-s0006.docx Whole-genome alignment of FDN strains and IBD-related strains.

Table S6 spectrum.02110-24-s0007.xlsx Virulence factors detected by ecolivf and vfdb in all tested strains’ chromosomes.

Table S7 spectrum.02110-24-s0008.docx Detailed information of plasmids from five FDN *E. coli* isolates.

Table S8 spectrum.02110-24-s0009.xlsx Annotated genes present only in FDN-4 and EC958, along with the genomic island in which each is located.

ASM does not own the copyrights to Supplemental Material that may be linked to, or accessed through, an article. The authors have granted ASM a non-exclusive, world-wide license to publish the Supplemental Material files. Please contact the corresponding author directly for reuse.
