# Supplementary figures and images for: Whole-genome analysis of five Escherichia coli strains isolated from focal duodenal necrosis in laying hens reveals genetic similarities to the E. coli O25:H4 ST131 strain
Source: Microbiol Spectr. 2025 Mar 31;13(5):e02110-24. doi: 10.1128/spectrum.02110-24 (PMC12054123; doi:10.1128/spectrum.02110-24)

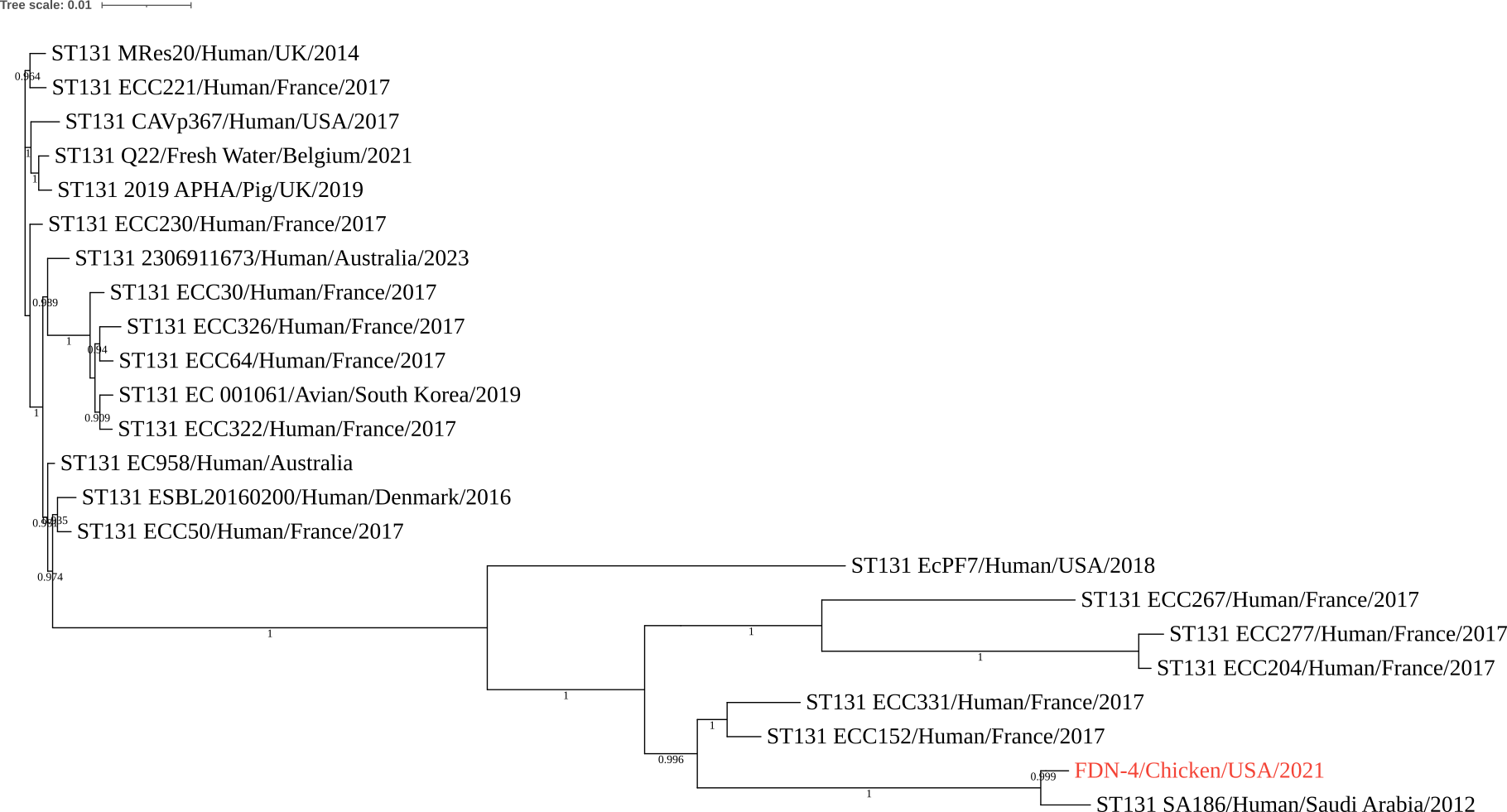
Supplementary Figure 1. Phylogenetic tree with FDN-4 and O25:H4 ST131 strains from Enterobase database

Supplement: Figure S1 — Phylogenetic tree with FDN-4 and O25:H4 ST131 strains from the Enterobase database. [file spectrum.02110-24-s0001.docx]
