# Supplementary material for: Whole-genome analysis of five Escherichia coli strains isolated from focal duodenal necrosis in laying hens reveals genetic similarities to the E. coli O25:H4 ST131 strain
Source: Microbiol Spectr. 2025 Mar 31;13(5):e02110-24. doi: 10.1128/spectrum.02110-24 (PMC12054123; doi:10.1128/spectrum.02110-24)
Supplement: Table S1 — E. coli strains included in the phylogenetic tree. [file spectrum.02110-24-s0002.docx]

Supplementary Table 1. *E. coli* strains included in phylogenetic tree. This table includes the accession numbers, host sources, collection years, countries of origin, and references for the *E. coli* strains used in the phylogenetic tree analysis.

| Strain | Accession # | Host | Collection Year | Country | Reference |
| --- | --- | --- | --- | --- | --- |
| AIEC 18I08 | [JANDAH010000001.1](https://www.ncbi.nlm.nih.gov/nuccore/JANDAH010000001.1) | Human | 2011/2012 | Spain | [Saitz Rojas, W. L.(2022)](https://pubmed.ncbi.nlm.nih.gov/36012279/) |
| AIEC 541 15 | [AJWQ01000001.1](https://www.ncbi.nlm.nih.gov/nuccore/AJWQ01000001.1) | Human | 2006 | USA | [Suzuki,H. (2012)](https://www.ncbi.nlm.nih.gov/nuccore/AJWQ01000001.1) |
| AIEC 5I01 | [JANCZY010000001.1](https://www.ncbi.nlm.nih.gov/nuccore/JANCZY010000001.1) | Human | 2011/2012 | Spain | [Saitz Rojas, W. L.(2022)](https://pubmed.ncbi.nlm.nih.gov/36012279/) |
| AIEC 9C01 | [JANCZQ010000001.1](https://www.ncbi.nlm.nih.gov/nuccore/JANCZQ010000001.1) | Human | 2011/2012 | Spain | [Saitz Rojas, W. L.(2022)](https://pubmed.ncbi.nlm.nih.gov/36012279/) |
| APEC O1 | [CP000468.1](https://www.ncbi.nlm.nih.gov/nuccore/CP000468.1) | Turkey | N/A | USA | [Johnson,T.J.(2007)](https://www.ncbi.nlm.nih.gov/pmc/articles/PMC1855855/) |
| APEC O18 | [CP006830.1](https://www.ncbi.nlm.nih.gov/nuccore/CP006830.1) | Chicken | N/A | USA | [Mangiamele,P.M. (2013)](https://www.ncbi.nlm.nih.gov/nuccore/CP006830.1) |
| APEC O2 211 | [CP006834.2](https://www.ncbi.nlm.nih.gov/nuccore/CP006834.2) | Chicken | N/A | USA | [Mangiamele,P.M. (2013)](https://www.ncbi.nlm.nih.gov/nuccore/CP006834.2) |
| APEC strain E9 | [CP104500.1](https://www.ncbi.nlm.nih.gov/nucleotide/CP104500.1?report=genbank&log$=nucltop&blast_rank=1&RID=2NBN9ZJE016) | Chicken | 2017 | USA | [Feng,A.(2022)](https://www.ncbi.nlm.nih.gov/nucleotide/CP104500.1?report=genbank&log$=nucltop&blast_rank=1&RID=2NBN9ZJE016) |
| EIEC O121 | [CP031910.1](https://www.ncbi.nlm.nih.gov/nuccore/CP031910.1) | Human | 2003 | Canada | [Cabral,J.(2017)](https://www.ncbi.nlm.nih.gov/nuccore/CP031910.1) |
| EIEC O96 H19 | [CP011416.1](https://www.ncbi.nlm.nih.gov/nuccore/CP011416.1) | Human | 2012 | Italy | [Strain,E.A.(2015)](https://pubmed.ncbi.nlm.nih.gov/26251502/) |
| EIEC.53638 | [NZ_AAKB02000001.1](https://www.ncbi.nlm.nih.gov/nuccore/NZ_AAKB02000001.1) | Human | N/A | USA | [Rasko,D.A.(20050](https://www.ncbi.nlm.nih.gov/nuccore/NZ_AAKB02000001.1) |
| EPEC E110019 | [CP035751.1](https://www.ncbi.nlm.nih.gov/nuccore/CP035751.1) | Human | N/A | USA | [Hazen,T.H(2019)](https://www.ncbi.nlm.nih.gov/pmc/articles/PMC6759289/) |
| ExPEC F11 | [CP076123.1](https://www.ncbi.nlm.nih.gov/nuccore/CP076123.1) | Human | N/A | Australia | [Forde,B.M. (2021)](https://www.ncbi.nlm.nih.gov/nuccore/CP076123.1) |
| FDN-4 | [CP158026.1](https://www.ncbi.nlm.nih.gov/nuccore/CP158026.1) | Chicken | 2021 | USA | From this study |
| FDN-9 | [CP158140.1](https://www.ncbi.nlm.nih.gov/nuccore/CP158140) | Chicken | 2021 | USA | From this study |
| FDN-11 | [CP158143.1](https://www.ncbi.nlm.nih.gov/nuccore/CP158143) | Chicken | 2021 | USA | From this study |
| FDN-24 | [CP158147.1](https://www.ncbi.nlm.nih.gov/nuccore/CP158147) | Chicken | 2021 | USA | From this study |
| FDN-50 | [CP158150.1](https://www.ncbi.nlm.nih.gov/nuccore/CP158150.1) | Chicken | 2021 | USA | From this study |
| IBD HM605 | [NZ_HE572566.1](https://www.ncbi.nlm.nih.gov/nuccore/NZ_HE572566.1) | Human | N/A | UK | [Clarke,D.J(2011)](https://pubmed.ncbi.nlm.nih.gov/21705601/) |
| IBD LF82 | [CU651637.1](https://www.ncbi.nlm.nih.gov/nuccore/CU651637.1) | Human | N/A | France | [Peyretaillade,E.(2010)](https://journals.plos.org/plosone/article?id=10.1371/journal.pone.0012714) |
| IBD NRG857 | [NC_017634.1](https://www.ncbi.nlm.nih.gov/nuccore/NC_017634.1) | Human | N/A | Germany | [Eaves-Pyles,T. (2008)](https://pubmed.ncbi.nlm.nih.gov/17900983/) |
| IBD UM146 | [NC_017632.1](https://www.ncbi.nlm.nih.gov/nuccore/NC_017632.1) | Human | N/A | USA | [Krause,D.O.(2010)](https://www.ncbi.nlm.nih.gov/pmc/articles/PMC3019814/) |
| K12 MG1655 | [U00096](https://www.ncbi.nlm.nih.gov/nuccore/U00096.3) | Human | 1922 | USA | [Blattner, F. R (1997)](https://www.science.org/doi/abs/10.1126/science.277.5331.1453?casa_token=y9jQ9qW6RaoAAAAA:J3-uWWLYm0u3jZB-8BFYXig8BiqioNwmywyzOIqgRswYh99b-I0gLLZnS1rSNkM46JEAljyFkoWbZg) |
| NMEC O18 | [CP007275.1](https://www.ncbi.nlm.nih.gov/nuccore/CP007275.1) | Human | N/A | USA | [Mangiamele,P.M.(2013)](https://www.ncbi.nlm.nih.gov/nuccore/CP007275.1) |
| ST131 CAVp367 | [CP145139.1](https://www.ncbi.nlm.nih.gov/nuccore/CP145139.1) | Human | 2017 | USA | [Sheppard,A.E. (2024)](https://www.ncbi.nlm.nih.gov/nuccore/CP145139.1) |
| ST131 EcPF7 | [NZ_CP054232.1](https://www.ncbi.nlm.nih.gov/nuccore/NZ_CP054232.1) | Human | 2018 | USA | [Sharon,B.M. (2020)](https://www.ncbi.nlm.nih.gov/nuccore/NZ_CP054232.1) |
| ST131 SA186 | [NZ_CP022730.1](https://www.ncbi.nlm.nih.gov/nuccore/NZ_CP022730.1) | Human | 2012 | Saudi Arabia | [Alghoribi,M.F.(2017)](https://www.ncbi.nlm.nih.gov/nuccore/NZ_CP022730.1) |
| ST131 2019 APHA | [NZ_CP051609.1](https://www.ncbi.nlm.nih.gov/nuccore/NZ_CP051609.1) | Pig | 2019 | UK | [Duggett,N.A.(2020)](https://www.ncbi.nlm.nih.gov/nuccore/NZ_CP051609.1) |
| ST131 EC958 | [NZ_HG941718.1](https://www.ncbi.nlm.nih.gov/nuccore/NZ_HG941718.1) | Human | N/A | Australia | [Forde,B.M.(2014)](https://pubmed.ncbi.nlm.nih.gov/25126841/) |
| STEC367 | [NZ_CP041429.1](https://www.ncbi.nlm.nih.gov/nuccore/NZ_CP041429.1) | Raw mutton | 2014 | China | [Yang,X.(2020)](https://pubmed.ncbi.nlm.nih.gov/31757694/) |
| UPEC 536 | [CP000247.1](https://www.ncbi.nlm.nih.gov/nuccore/CP000247.1) | Human | N/A | Germany | [Hochhut,B.(2006)](https://pubmed.ncbi.nlm.nih.gov/16879640/) |
| UPEC CFT073 | [AE014075.1](https://www.ncbi.nlm.nih.gov/nuccore/AE014075.1) | Human | 1990 | USA | [Welch,R.A.(2002)](https://pubmed.ncbi.nlm.nih.gov/12471157/) |
| UPEC UTI98 | [CP000243.1](https://www.ncbi.nlm.nih.gov/nuccore/CP000243.1) | Human | N/A | USA | [Chen,S.L.(2006)](https://pubmed.ncbi.nlm.nih.gov/16585510/) |
