# Supplementary material for: Whole-genome analysis of five Escherichia coli strains isolated from focal duodenal necrosis in laying hens reveals genetic similarities to the E. coli O25:H4 ST131 strain
Source: Microbiol Spectr. 2025 Mar 31;13(5):e02110-24. doi: 10.1128/spectrum.02110-24 (PMC12054123; doi:10.1128/spectrum.02110-24)
Supplement: Table S5 — Whole-genome alignment of FDN strains and IBD-related strains. [file spectrum.02110-24-s0006.docx]

Supplementary Table 5. Whole genome alignment of FDN strains and IBD related strains.

|  | FDN-4 | FDN-9 | FDN-11 | FDN-24 | FDN-50 | LF82 | HM605 | UM146 |
| --- | --- | --- | --- | --- | --- | --- | --- | --- |
|  | % Query cover/ % Identity | | | | | | | |
| FDN-4 |  | 77/97.8 | 78/97.6 | 77/97.8 | 77/98.1 | 83/99.16 | 84/98.93 | 86/98.91 |
| FDN-9 | 77/97.8 |  | 91/98.9 | 99/100 | 90/98.8 | 83/97.83 | 85/97.9 | 84/97.88 |
| FDN-11 | 78/97.6 | 91/98.9 |  | 91/98.9 | 91/99.5 | 82/98.09 | 84/97.62 | 83/97.6% |
| FDN-24 | 77/97.8 | 99/100 | 91/98.9 |  | 89/98.8 | 83/97.83 | 85/ 7.9 | 84/97.88 |
| FDN-50 | 77/98.1 | 90/98.8 | 91/99.5 | 89/98.8 |  | 81/98.12 | 83/98.01 | 83/98.07 |
