# Supplementary material for: Whole-genome analysis of five Escherichia coli strains isolated from focal duodenal necrosis in laying hens reveals genetic similarities to the E. coli O25:H4 ST131 strain
Source: Microbiol Spectr. 2025 Mar 31;13(5):e02110-24. doi: 10.1128/spectrum.02110-24 (PMC12054123; doi:10.1128/spectrum.02110-24)
Supplement: Table S7 — Detailed information of plasmids from five FDN E. coli isolates. [file spectrum.02110-24-s0008.docx]

|  | Plasmid size (bp) | Plasmidfinder/plasmidtype | BLAST (Query cover/Per, ident.) | Bacteria Species | Original Source | Original Country | Year |
| --- | --- | --- | --- | --- | --- | --- | --- |
| [pFDN4-1](https://www.ncbi.nlm.nih.gov/nuccore/CP158027.1) | 182,828 | IncFIB/IncFIC | [PRJNA953191](https://www.ncbi.nlm.nih.gov/nucleotide/CP134377.1?report=genbank&log$=nucltop&blast_rank=1&RID=VUEAXZ7R013) (95%/99.9%) | *E. coli* | Chicken meat | Brazil | 2012 |
| [pFDN4-2](https://www.ncbi.nlm.nih.gov/nuccore/CP158028.1) | 35,593 | IncX1 | [PRJEB42440](https://www.ncbi.nlm.nih.gov/nucleotide/OW967202.1?report=genbank&log$=nucltop&blast_rank=1&RID=W2B13G8P016) (100%/99.96%) | *Klebsiella pneumoniae* | Human | Spain | 2022 |
| [pFDN4-3](https://www.ncbi.nlm.nih.gov/nuccore/CP158028.1) | 7,419 | Not identified | [PRJNA1013080](https://www.ncbi.nlm.nih.gov/nucleotide/CP134662.1?report=genbank&log$=nucltop&blast_rank=1&RID=W2B5E3PT016) (98%/99.97%) | *E. coli* | Gull | France | 2022 |
| [pFDN9-1](https://www.ncbi.nlm.nih.gov/nuccore/CP158141) | 148,663 | IncFIB/IncFII | [PRJNA481171](https://www.ncbi.nlm.nih.gov/nucleotide/CP031107.1?report=genbank&log$=nucltop&blast_rank=1&RID=W2BMGFK9016) (83%/99.75%) | *E. coli* | Giant panda | China | 2018 |
| [pFDN9-2](https://www.ncbi.nlm.nih.gov/nuccore/CP158142) | 88,248 | IncI1-I | [PRJEB24625](https://www.ncbi.nlm.nih.gov/nucleotide/LT985268.1?report=genbank&log$=nucltop&blast_rank=1&RID=WU26CVKD013) (94%/98.74%) | *E. coli* | NA | France | 2018 |
| [pFDN11-1](https://www.ncbi.nlm.nih.gov/nuccore/CP158144) | 135,559 | IncFIB/IncFIC | [PRJNA384272](https://www.ncbi.nlm.nih.gov/nucleotide/CP021198.1?report=genbank&log$=nucltop&blast_rank=1&RID=WU346W7901N) (69%/99.8%) | *E. coli* | Human | China | 2012 |
| [pFDN11-2](https://www.ncbi.nlm.nih.gov/nuccore/CP158145) | 48,395 | Not identified | [PRJNA843104](https://www.ncbi.nlm.nih.gov/nucleotide/CP098182.1?report=genbank&log$=nucltop&blast_rank=1&RID=WU3BRZ8301N) (100%/99.99%) | *E. coli* | Human | South Korea | 2017 |
| [pFDN11-3](https://www.ncbi.nlm.nih.gov/nuccore/CP158146) | 32,596 | IncX1 | [PRJNA605147](https://www.ncbi.nlm.nih.gov/nucleotide/CP057914.1?report=genbank&log$=nucltop&blast_rank=1&RID=WU3GTADF013) (100%/99.94%) | *E. coli* | Sheep | United Kingdom | 2017 |
| [pFDN24-1](https://www.ncbi.nlm.nih.gov/nuccore/CP158148) | 148,663 | IncFIB/IncFII | [PRJNA481171](https://www.ncbi.nlm.nih.gov/nucleotide/CP031107.1?report=genbank&log$=nucltop&blast_rank=1&RID=WU450VK401N) (83%/99.75%) | *E. coli* | Giant panda | China | 2018 |
| [pFDN24-2](https://www.ncbi.nlm.nih.gov/nuccore/CP158149) | 88,172 | IncI1-I | [SAMN14227211](https://www.ncbi.nlm.nih.gov/nucleotide/KU932027.1?report=genbank&log$=nucltop&blast_rank=1&RID=WU4BV8Z701N) (97%/98.76%) | *E. coli* | Human | Finland | 2014 |
| [pFDN50-1](https://www.ncbi.nlm.nih.gov/nuccore/CP158151.1) | 158.974 | IncFIB/IncFII | [PRJNA230969](https://www.ncbi.nlm.nih.gov/nucleotide/CP070400.1?report=genbank&log$=nucltop&blast_rank=1&RID=WU4X2R8001N) (79%/99.65%) | *E. coli* | Chicken | Romania | 2016 |
| [pFDN50-2](https://www.ncbi.nlm.nih.gov/nuccore/CP158152.1) | 89,593 | IncI1-I | [PRJNA485472](https://www.ncbi.nlm.nih.gov/nucleotide/CP032445.1?report=genbank&log$=nucltop&blast_rank=1&RID=WU69CHU5016) (99%/98.87%) | *Salmonella enterica* | Bovine | United States | 2012 |
| [pFDN50-3](https://www.ncbi.nlm.nih.gov/nuccore/CP158153.1) | 36,542 | IncX4 | [PRJEB42440](https://www.ncbi.nlm.nih.gov/nucleotide/OW967979.1?report=genbank&log$=nucltop&blast_rank=1&RID=WU6GDR2F016) (96%/99.98%) | *E. coli* | Human | Spain | 2022 |

Supplementary Table 7. Detailed information of plasmids from 5 FDN *E. coli* isolates. BALSTn was used on all plasmid sequences to find the most genetic similar reference (the species, source, country and year) in the NCBI database.
